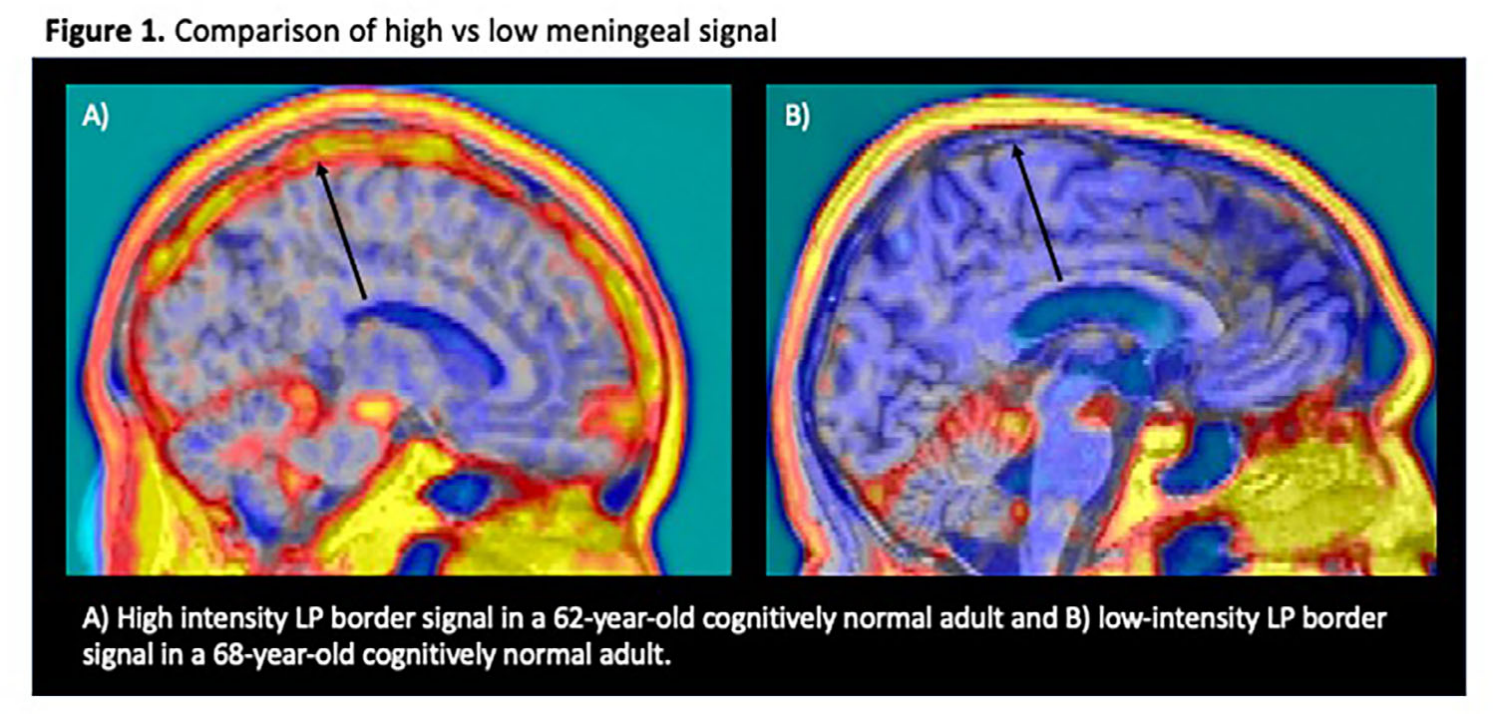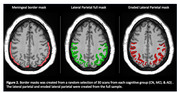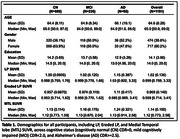# Reducing contamination from off‐target binding in the meninges in PI‐2620 tau PET scans

**DOI:** 10.1002/alz.083984

**Published:** 2025-01-03

**Authors:** Maxwell W Hand, Victoria R Tennant, Noelle Lee, Meral A Tubi, Arthur W. Toga, Sid E. O'Bryant, Bradley T. Christian, Meredith N Braskie

**Affiliations:** ^1^ Stevens Neuroimaging and Informatics Institute, Marina del Rey, CA USA; ^2^ Stevens Neuroimaging and Informatics Institute, Los Angeles, CA USA; ^3^ Mark and Mary Stevens Neuroimaging and Informatics Institute, Keck School of Medicine, University of Southern California, Los Angeles, CA USA; ^4^ Imaging Genetics Center, Mark and Mary Stevens Neuroimaging and Informatics Institute, Keck School of Medicine, University of Southern California, Marina del Rey, CA USA; ^5^ University of North Texas Health Science Center, Fort Worth, TX USA; ^6^ University of Wisconsin‐Madison, Madison, WI USA

## Abstract

**Background:**

Positron emission tomography (PET) tau tracer PI‐2620 frequently shows off‐target meningeal binding (**Figure 1**). Of standard regions of interest, only lateral parietal (LP) is contaminated by this. We compare the standardized uptake value ratio (SUVR) in the LP before and after eroding the LP mask to remove meningeal contamination.

**Method:**

We evaluated 1191 older adults from the Health & Aging Brain Study‐Health Disparities with available [18F]‐PI‐2620‐PET scans (**Table 1**). We created: 1) a FreeSurfer‐derived LP mask 2) an eroded LP mask, and 3) in a subset of 90 participants (30 participants from each cognitive group) an LP border mask capturing meningeal signal (**Figure 2**).

To create the eroded LP mask, we eroded a full brain mask (FSL erode kernel sphere 3) and multiplied it by the Freesurfer‐derived LP mask. To create the LP border mask we dilated each participant’s LP mask (kernel box 6), subtracted the brain mask, and thresholded at 0. We used linear regression to examine relationships between: 1) original and eroded LP SUVR and 2) LP border tau‐binding and SUVR change (original LP SUVR minus eroded LP SUVR). We also related eroded and non‐eroded LP signal to 1) medial temporal SUVR, which is not strongly affected by meningeal signal and 2) age.

**Result**

The eroded LP SUVRs and original LP SUVRs were highly correlated (*β* = 0.93; p<0.01). Age was associated with LP SUVR after (*β* = 0.15; *p*<0.01), but not before (*β* = 0.026; *p* = 0.37), LP mask erosion. Higher medial temporal lobe SUVR was associated with higher LP SUVR both before and after (both *p*<0.01) erosion of the LP mask. In the 90 participants with LP border masks, higher LP border signal was associated with greater LP SUVR change after erosion (*β* = 0.048, *p*<0.01).

**Conclusion**

SUVR contamination in the LP due to off‐target PI‐2620 binding was reduced using mask erosion. Greater change in SUVR was associated with higher initial contamination (indicated by more off‐target signal contiguous to the LP region). Mask erosion also strengthened the relationship between LP SUVR and age, suggesting that removing contamination from the meninges helped highlight true tau signal.